# A comprehensive analysis of trends and determinants of HIV/AIDS knowledge among the Bangladeshi women based on Bangladesh Demographic and Health Surveys, 2007–2014

**DOI:** 10.1186/s13690-017-0228-2

**Published:** 2017-09-28

**Authors:** Md. Tuhin Sheikh, Md. Nizam Uddin, Jahidur Rahman Khan

**Affiliations:** Institute of Statistical Research and Training, University of Dhaka, Shahbagh, Dhaka, 1000 Bangladesh

**Keywords:** HIV/AIDS awareness, Women, Mixed model, Bangladesh

## Abstract

**Background:**

South-Asian countries are considered to be a potential breeding ground for HIV epidemic. Although the prevalence of this incurable disease is low in Bangladesh, women still have been identified as more vulnerable group. The aim of this study is to assess the knowledge about HIV/AIDS: its trends and associated factors among the women in Bangladesh.

**Methods:**

We analysed the nationally representative repeatedly cross-sectional Bangladesh Demographic and Health Surveys (BDHSs) data: 2007, 2011, and 2014. These data were clustered in nature due to the sampling design and the generalized mixed effects model is appropriate to examine the association between the outcome and the explanatory variables by adjusting for the cluster effect.

**Results:**

Overall, women’s knowledge about HIV/AIDS has been decreasing over the years. Education plays the leading role and secondary-higher educated women are 6.6 times more likely to have HIV/AIDS knowledge. The likelihood of knowledge is higher among the women who had media exposure (OR: 1.6) and knowledge on family planning (OR: 2.3). A rural-urban gap is noticed in women’s knowledge about HIV/AIDS and significant improvement has been observed among the rural and media exposed women. Results reveal that age, region, religion, socio-economic status, education, contraceptive use have significant (*p*<0.01) effects on women’s knowledge about HIV/AIDS.

**Conclusion:**

This study recommends to emphasis more on women’s education, media exposure, and family planning knowledge in strengthening women’s knowledge about HIV/AIDS. In addition, residence specific programs regarding HIV/AIDS awareness also need to be prioritized.

## Background

Among the incurable infectious diseases, acquired immune deficiency syndrome (AIDS) caused by the infection of humane immunodeficiency virus (HIV) has become a major global health problem in recent years. According to the UNAIDS [1], there were 36.7 million people living with HIV in 2015, which is 3.4 million higher than those of in 2010. In Asia and Pacific region, there were 5.1 million people living with HIV in 2015 [2], of which the South-Asian (SA) countries: China, India, and Indonesia account for about 75% of the total number of people living with HIV in this region [3]. In Bangladesh-a SA country, the prevalence of HIV is low (less than 0.1%) [4], which steadily increased since 1989 [5]. The reported number of people with HIV in Bangladesh increased by more than 300% (from 1207 in 2007 to 3674 in 2014) in seven years [6]. The recent estimates of the number of people living with HIV in Bangladesh in 2015 is about 9600, of which about 3200 are women aged 15 years and above [7]. Thus, Bangladesh with her low prevalence of HIV/AIDS, possesses a high risk of rapid spread of HIV/AIDS [5, 8–10].

There are many potential factors that are attributable to this increased risk of HIV infection and/or transmission: geographical and cultural proximity to India and Myanmar-two severely affected countries [8, 11], poverty, gender inequity, high levels of transitional sex [12], mobility of boatmen across the border area [13], and especially, the low-level knowledge about HIV/AIDS. With the vision of reducing the risk of HIV infection and transmission, we should concentrate on these potential factors, however, many of these factors are often linked with the country’s health, demography, economy, politics, etc., which are not malleable enough to change or improvement. Instead, major concentration could be given on increasing the level of knowledge about HIV/AIDS, since the causes of HIV infection are known and can be escaped by being knowledgeable about HIV/AIDS. In the context of Bangladesh, the percentage of married women and married men with knowledge about HIV/AIDS were 67% and 87%, respectively, in 2007 [14]. This percentage increased only by 2% for married women and 1% for married men, respectively over the years 2007–2011 [15]. In 2014, Bangladesh Demographic and Health Survey (BDHS) identified female population as more vulnerable group than male population and observed that about 70% of the married women are knowledgeable about HIV/AIDS, which is very similar to that of documented in 2011 [6]. Accordingly, many studies [16, 17] reported that the level of knowledge among the men is higher compared to the women in Bangladesh. Moreover, the women bear the heavier onus of the consequences of the disease due to their standing in a less advantaged socio-economic position, limited access to sexually, and reproductive health care [18], and subsequently, women are considered to be more vulnerable to HIV infection and transmission [10]. In addition, the perception among the women in Bangladesh about HIV/AIDS is often contaminated with myths, facts, and rumors [19], which further contribute to HIV infection and/or transmission.

In this critical condition, to control HIV infection and/or transmission, preventive measures (e.g. increasing the level of knowledge) for women could be effective, which has been recommended in earlier studies [16, 20, 21]. Since any effective vaccine to completely cure from HIV/AIDS is not available yet [22], spreading correct knowledge about HIV/AIDS should be the very first step to raise awareness about HIV/AIDS. Lack of knowledge about HIV/AIDS is usually positively associated with misconception, confusion, social stigma, poor sex behavior [23], which contribute to the increase in HIV infection and transmission. Increasing women’s knowledge about HIV/AIDS will facilitate long-term controlling of HIV/AIDS epidemic [17] and will still be effective even when there is limited/poor healthcare facilities. Assessing the current scenario of women’s knowledge status in Bangladesh and identifying the associated factors will be helpful for government and non-government organizations to develop more structured and specific target program regarding HIV/AIDS prevention.

In this regard, Khan [21] investigated the adolescents married women (10–19 years) in Bangladesh and reported female education, media use, and condom use as potential predictors of women’s knowledge about HIV/AIDS. Rahman, M.S. and Rahman, M.L. [16] studied married women of wider age group (15–49 years) and identified the use of media as a strong tool to spread HIV knowledge and, also reported socio-economic status as an important factor. Likewise, Yaya et al. [17] studied a sample of ever married women in Bangladesh and demonstrated a positive association between the women’s knowledge and their respective husbands’ increasing level of education. Although there have been notable research works conducted earlier to assess the knowledge status of married women in Bangladesh, most studies focused on a particular study period. There are only few studies [24] that examined the trends and determinants of knowledge about HIV/AIDS among the married women in Kenya over the years 1993–2009. Studying the trends and determinants of women’s knowledge will disclose more window about the changing behavior of the associated factors and their varying effects over time. To best of our knowledge, no earlier studies in Bangladesh examined the trends and determinants associated with the knowledge about HIV/AIDS among the married women in Bangladesh.

The main goal of the study is to analyse the pooled data from the repeatedly cross-sectional BDHSs of the period 2007–2014, and investigate the factors associated with the married women’s knowledge about HIV/AIDS in Bangladesh and their trends over time. Mixed effects model is used to analyse BDHS data to have greater insight into the data mechanism. This study will help the government and policy makers to evaluate the present scenario of knowledge about HIV/AIDS among the women in Bangladesh, and the trend analysis will help to investigate the pattern of changes in the determinants of knowledge about HIV/AIDS. We expect this study will help in constructing necessary programs that might contribute to control HIV infection or AIDS disease in Bangladesh.

## Methods

### Sampling design

The data for this study is extracted from the Bangladesh Demographic and Health Surveys (BDHSs) of 2007, 2011, and 2014. These nationally representative cross-sectional surveys are conducted in collaboration with National Institute of Population Research and Training (NIPORT), ICF International, USA, and Mitra & Associates. The sampling design for these surveys is comprised of two-stage stratified cluster sampling scheme. The sampling frame for the survey is a complete list of enumeration areas (EAs), which are either a village or a part of a village or a group of villages. In the first stage of the sampling, EAs (clusters) are selected using probability proportional to size (PPS) sampling method, where 600 clusters were selected in all BDHSs 2007, 2011, and 2014, respectively. In the second stage, an equal probability systematic sampling method was used to draw on average 30 households from each cluster. Finally, a total of 10996, 17749, and 17863 households were selected in the BDHS 2007, 2011, and 2014, respectively.

### Data and variables

The Bangladesh Demographic and Health Survey (BDHS) collects data on demographics, fertility, mortality, nutrition, awareness and attitude towards HIV/AIDS, etc., although the collection of data is often subject to the objective and vision of the year of survey. To investigate the trends and determinants of the knowledge about HIV/AIDS among the women in Bangladesh, we pooled the last three survey data of BDHSs (2007–2014) and focused on the ever married sample to investigate our hypothesis. The main variable of interest is the knowledge status of married women about HIV/AIDS, which is extracted by asking the women whether they have heard about HIV/AIDS or not. To determine the effects of associated factors on the knowledge about HIV/AIDS, the relevant covariates are analysed that are reported in the earlier studies [5, 19, 21, 25, 26], depending on their availability in the BDHSs data. For instance, respondent’s age (“15–19”, “20–24”, “25–29”, “30–49”), respondent’s and husband’s highest level of education (“No education”, “Primary”, “Secondary or Higher”), division (“Barisal”, “Chittagong”, “Dhaka”, “Khulna”, “Rajshahi”, “Sylhet”), residence (“Rural”, “Urban”), Religion (“Islam”, “Others”), socio-economic status (“Poor”, “Middle”, “Rich”). The variable on contraceptive use is categorized into two, “Yes” if any method (condom or others) and “No” for no method. Media use is also categorized as “Yes” for those respondents who either watch television or listen to radio or read newspaper. And, a variable on family planning knowledge is categorized as “Yes” for the respondents who heard about family planning through media and “No” otherwise. For BDHS 2011 and 2014, we have merged Rangpur and Rajshahi divisions as Rajshahi division for matching with BDHS 2007. There are some other important variables, e.g. information of HIV incidence, women’s knowledge about HIV programs and sources of information, etc. which could help explaining women’s knowledge about HIV/AIDS, however, due to unavailability of such information in BDHS data we could not investigate these variables. For details of the design and survey questions regarding the data, we would refer the BDHS report of 2014 [6].

### Model

Let *Y*_*ij*_ (*i*=1,…,*N*; *j*=1,…,*n*_*i*_) be the binary response of knowledge about HIV/AIDS for the *j*th woman under the *i*th EA (cluster), which takes the value of 1 if the women heard about HIV/AIDS and 0 otherwise. In the BDHSs pooled data, women within the same cluster may share some similar characteristics, facilities, and/or commodities, which may result in correlated responses within the clusters. Thus the data have a hierarchical structure, women are nested within the clusters. To analyse clustered data with hierarchical structure, regular statistical tool, e.g. simple logistic regression often unable to adjust for the variation due to cluster and fails to capture the association among the responses within clusters. Disregarding the variation due to cluster and cluster-specific association among the responses results in incorrect standard errors that compromise the efficiency of the model parameters [27]. To model clustered binary outcome, generalized linear mixed effects model (GLMM) is often used, which is comprised of fixed and random effects components. The fixed effects explain the population average evolution in response to covariates, and random effects explain the cluster-specific evolution. The GLMM with logit link is defined as

1 $$ \text{logit}(p_{ij})=\mathbf{x}'_{ij} \boldsymbol{\beta}+u_{i},  $$

where *p*_*ij*_ denotes the probability that a woman have heard about HIV/AIDS i.e. *P* *r*(*y*_*ij*_=1), **x** *ij*′ is the vector of observed covariates, ***β*** is the vector of regression parameters, and the random effects or residuals at the cluster level, *u*_*i*_s are assumed to follow normal distribution with zero mean and constant variance $\sigma ^{2}_{u}$. That is, ***β***s explain the effects of the covariates averaged over the clusters and random effects *u*_*i*_ adjust for the cluster specific evolution of the mean response. To determine the extent to which the respondent’s knowledge status about HIV/AIDS is clustered within clusters, the variance estimates at the cluster level and the individual level are used to evaluate the intra-cluster correlation (ICC), which is defined as

$$\rho=\frac{\sigma^{2}_{u}}{\sigma^{2}_{u}+\sigma^{2}_{e}}, $$

where $\sigma ^{2}_{e}$ is the total variance at individual level. In hierarchical data structure, the residuals at individual level are assumed to follow standard logistic regression with zero mean and variance $\sigma ^{2}_{e}=\pi ^{2}/3$ [27]. The value of *ρ* is close to zero for a clustered data with no cluster variation, on the other hand, *ρ*>0 indicates the average correlation among the binary responses within clusters. The estimation of the model parameters defined in (1) is evaluated by maximization of the likelihood function. For more detail about the parameter estimation, we refer the reader the references, Fitzmaurice et al. [28] and Powers and Xie [27].

## Results

Table 1 presents the descriptive statistics of the variables considered in the analysis, across the survey years 2007–2014. The descriptive measures show that the percentage of women with knowledge about HIV/AIDS slightly increased for the women aged 25–49 years and decreased for the women aged 15–24 years, of which young married women (15–19 years) show a more steeper decline. The rationale behind this change is that the young married women have already been identified as at risk group who lack maturity and often get limited access to health-related discussion, especially, sex-related health problems. We observe that the frequency of married women aged 15–19 years increased from 1051 in 2007 to 1462 in 2014, which have contributed to the decrease in the percentage of knowledgeable married women (15–19 years) over the three survey years. For all three of the survey years, the percentages of women with knowledge about HIV/AIDS are highest and lowest for the age group of 20–24 years and 30–49 years, respectively.

**Table 1:** Distribution of Bangladeshi married women with knowledge about HIV/AIDS by various demographic and socio-economic variables(Bangladesh Demographic and Health Surveys, 2007–2014)

	Frequency (%) of women with HIV/AIDS knowledge		
Variables	2007	2011	2014
Age			
15-19	1051 (77.97)	1441 (75.41)	1462 (72.27)
20-24	1737 (79.9)	2724 (78.82)	2456 (77.7)
25-29	1436 (74.25)	2562 (75.64)	2610 (78.07)
30-49	3463 (62.52)	5729 (63.69)	6065 (64.96)
Religion			
Islam	6953 (70.07)	11070 (70.25)	11454 (70.99)
Others	733 (68.44)	1386 (69.61)	1138 (65.89)
Respondent’s education			
No education	1576 (44.71)	1876 (40.53)	1713 (40.73)
Primary	2197 (67.23)	3401 (64.22)	3320 (63.53)
Secondary or higher	3911 (93.14)	7179 (91.76)	7560 (89.67)
Husband’s education			
No education	1811 (50.28)	2421 (46.69)	2445 (48.3)
Primary	1918 (66.57)	3193 (66.63)	3193 (65.77)
Secondary or higher	3950 (87.82)	6836 (88.06)	6952 (87.52)
Socio-economic status			
Poor	1798 (47.69)	3106 (48.59)	3313 (50.11)
Middle	1382 (65.97)	2390 (70.23)	2600 (71.8)
Rich	4507 (87.86)	6960 (87.5)	6680 (87.54)
Working status			
No	5567 (71.75)	10726 (69.74)	8779 (71.76)
Yes	2119 (65.54)	1730 (73)	3810 (67.75)
Contraceptive use			
No	3468 (64.61)	5028 (66.95)	4955 (67.04)
Yes	4219 (74.98)	7428 (72.55)	7638 (72.94)
Media use			
No	4422 (61.17)	8736 (64.06)	9625 (65.52)
Yes	3263 (86.78)	3702 (90.49)	2952 (93.77)
Family planning knowledge			
No	3902 (57.04)	7981 (62.57)	9227 (64.96)
Yes	3785 (91.12)	4475 (89.63)	3362 (92.03)
Residence			
Urban	3549 (85.52)	5169 (83.65)	5125 (83.1)
Rural	4138 (60.45)	7287 (62.98)	7468 (63.85)
Division			
Barisal	957 (66.55)	1510 (73.66)	1570 (73.3)
Chittagong	1311 (67.51)	2024 (70.67)	2041 (71.24)
Dhaka	1795 (76.71)	2324 (75.9)	2403 (77.69)
Khulna	1375 (80.36)	2144 (81.21)	2035 (78.85)
Rajshahi	1364 (65.58)	3156 (62.53)	3225 (63.95)
Sylhet	885 (59.64)	1298 (62.22)	1319 (61.66)

In the extracted pooled data, majority of the respondents (about 90%) were Muslim, of which about 70% women have knowledge about HIV/AIDS over the last three survey years. The higher percentage of educated women (primary or secondary/higher) have knowledge about HIV/AIDS compared to women with no education, for all three of the survey years. Similar to respondent’s education, a higher percentage of women have HIV/AIDS knowledge whose husbands have a higher level of educational attainment. Table 1 shows that the women from upper socio-economic class have a higher percentage of knowledgeable women about HIV/AIDS. In particular, the percentage of rich women with HIV/AIDS knowledge has been uniform (about 88%), while for the poor and middle-class women, the percentage increased by 2% (48 to 50%) and 6% (66 to 72%), respectively over the years 2007–2014. Women’s knowledge about HIV/AIDS varies for their working status, surprisingly, except for 2011, the percentage of working women having knowledge about HIV/AIDS are found to be lower in 2007 and 2014.

The percentage of knowledgeable women who use contraceptive during sexual intercourse is found to be higher compared to women who use no contraceptive, with the latter group shows a slightly increasing pattern over the years 2007–2014. Conversely, for the women who use contraceptive, the percentage of having knowledge about HIV/AIDS slightly decreased over the last three survey years. The percentage of knowledgeable women who have media exposure increased 9% (87 to 94%) over the years 2007–2014. Similarly, a higher percentage of women with family planning knowledge have knowledge about HIV/AIDS compared to women with no knowledge of family planning. The descriptive measures show that the percentage of knowledgeable women about HIV/AIDS changed disproportionately in urban and rural areas over the years. Although the rural-urban gap is declining over the years, the percentage of urban women having HIV/AIDS knowledge decreased, while the percentage increased for rural women. Khulna and Sylhet divisions have the highest and lowest percentages of women with knowledge about HIV/AIDS, respectively, for all three of the survey years.

Table 2 shows the results derived from generalized linear mixed effects model for assessing the association between the knowledge about HIV/AIDS among the Bangladeshi married women and different socio-demographic, socio-economic, and health-related factors. The results show that the age of women has a significant effect on their knowledge about HIV/AIDS. Particularly, women aged 20–29 years are more likely, while women aged 30 years or above are less likely to have knowledge about HIV/AIDS compared to women aged 15–19 years.

**Table 2:** Estimates and standard errors (SEs) of generalized linear mixed effects model parameters for the knowledge about HIV/AIDS among the women in Bangladesh (Bangladesh Demographic and Health Surveys, 2007–2014)

Variable	$\hat {\beta }$	OR: $\exp (\hat {\beta })$	SE	*p*-value
Age (ref: 15–19)				
20–24	0.309	1.362	0.073	<0.01
25–29	0.297	1.345	0.077	<0.01
30–49	-0.107	0.898	0.064	0.092
Religion (ref: Islam)				
Others	-0.209	0.811	0.053	<0.01
Respondent’s education (ref: No education)				
Primary	0.708	2.030	0.032	<0.01
Secondary or Higher	1.881	6.560	0.043	<0.01
Husband’s education (ref: No education)				
Primary	0.173	1.189	0.032	<0.01
Secondary or Higher	0.631	1.880	0.037	<0.01
Socio-economic status (ref: Poor)				
Middle	0.350	1.419	0.035	<0.01
Rich	0.741	2.098	0.039	<0.01
Working status (ref: No)				
Yes	0.089	1.093	0.033	<0.01
Contraceptive use (ref: No)				
Yes	0.245	1.278	0.092	<0.01
Media use (ref: No)				
Yes	0.465	1.592	0.067	<0.01
Family planning knowledge (ref: No)				
Yes	0.840	2.317	0.106	<0.01
Residence (ref: Urban)				
Rural	-0.932	0.394	0.105	<0.01
Division (ref: Barisal)				
Chittagong	-0.292	0.747	0.084	<0.01
Dhaka	0.277	1.319	0.084	<0.01
Khulna	0.570	1.769	0.088	<0.01
Rajshahi	-0.359	0.699	0.077	<0.01
Sylhet	-0.304	0.738	0.089	<0.01
Survey year (ref: 2007)				
2011	-0.193	0.825	0.107	0.073
2014	-0.197	0.821	0.107	0.065
Residence × Survey year				
Rural:2011	0.328	1.388	0.127	0.010
Rural:2014	0.370	1.448	0.127	<0.01
Media use × Survey year				
Yes:2011	0.088	1.092	0.095	0.351
Yes:2014	0.270	1.310	0.109	0.013
Residence × Contraceptive use				
Rural:Yes	-0.124	0.883	0.061	0.041
Respondent’s age × Contraceptive use				
20–24:Yes	-0.013	0.987	0.102	0.895
25–29:Yes	0.054	1.055	0.103	0.601
30–49:Yes	0.180	1.197	0.086	0.037
Respondent’s age × FPK				
20–24:Yes	0.243	1.275	0.141	0.085
25–29:Yes	0.379	1.460	0.144	<0.01
30–49:Yes	0.178	1.195	0.117	0.127
Constant	-0.313	0.732	0.124	0.012
Variance of random effect $(\hat {\sigma ^{2}_{u}})$	0.477			
ICC $(\hat {\rho })$	0.137			

Table 2 shows that the religion of women is also found to have a significant effect on women’s knowledge about HIV/AIDS. We found Muslim women are 20% more likely to have knowledge about HIV/AIDS compared to women of other religions (Hinduism, Christianity, etc.). As expected, there exists a positive association between women’s highest level of education and their knowledge about HIV/AIDS. For instance, the odds of women with primary and secondary/higher education levels are 1.2 and 1.9, respectively, to the women with no education. We found similar results for the husband’s education, i.e., the likelihood of women’s knowledge about HIV/AIDS among the women increases with the increasing attainment of their respective husband’s education. It is found that the knowledge about HIV/AIDS among the women is significantly related to their socio-economic status, with a higher level of socio-economic status indicates an increased likelihood of knowledge about HIV/AIDS. Particularly, women from middle and rich class are 1.4 and 2.1 times more likely to have knowledge about HIV/AIDS, respectively, compared to poor women.

Working women are 9% more likely to have knowledge about HIV/AIDS compared to the women with no working status. Contraceptive use during sex is one of the most convenient and easy ways to prevent HIV infection. The results show that the women using contraceptive during sex are more likely to have knowledge about HIV/AIDS (OR: 1.28). The effect of media use on the knowledge about HIV/AIDS is quantified through a composite index of watching television or listening radio or reading newspaper. The odds of women with media exposure having knowledge about HIV/AIDS is about 1.6 times higher compared to women without media exposure. Similarly, for women who have family planning knowledge, the likelihood of having knowledge about HIV/AIDS is found to be higher. Type of residence has significant effect on explaining the likelihood of women’s knowledge about HIV/AIDS. It is found that women living in urban areas have a higher likelihood of having knowledge about HIV/AIDS compared to women living in rural areas. The knowledge about HIV/AIDS among the women is found to significantly vary over the divisions of Bangladesh. Comparing with respect to Barisal division, respondents from both Dhaka and Khulna division are 1.3 and 1.8 times more likely, respectively, of having knowledge about HIV/AIDS. Whereas, women from Chittagong, Rajshahi, and Sylhet division have 25%, 30%, and 27% lower likelihood of having knowledge about HIV/AIDS, respectively, than that of the women from Barisal division.

To have the true picture of the effects of the determinants on the women’s knowledge about HIV/AIDS, we adjusted for the possible interaction effects in the analysis. The results show that the effect of residence type is different at different levels of the survey year and contraceptive use. The rural women who use contraceptive are 0.88 times less likely to have knowledge about HIV/AIDS. Also, among the rural women, the likelihood of having knowledge about HIV/AIDS shows an increasing trend over the years. The similar increasing trend has been found for the women who got media exposure over the years. The effect of age on the women’s knowledge has also been found to vary significantly over the levels of contraceptive use and family planning knowledge status. The likelihood of having knowledge about HIV/AIDS is 1.20 times more among the 30–49 years aged women who use contraceptives. Whereas, the family planning knowledge has a higher impact on the knowledge about HIV/AIDS among the women of all the age groups except for 30–49 years compared to women aged 15–19 years. Finally, the intra-cluster correlation (ICC) is found to be 0.137, which implies 13.7% of the variation in response is due to variation between clusters.

## Discussion

In this study, nationally representative pooled data from the Bangladesh Demographic and Health Survey (BDHS) of the years 2007, 2011, and 2014, are analysed to examine the trends and determinants of knowledge about HIV/AIDS among the married women (15–49 years) in Bangladesh. This study serves two important aspects through mixed model analysis of the data. First, analysing the determinants as well as their changes in pattern provides more insight to evaluate the present scenario of HIV/AIDS knowledge among the women compared to previous years and identify specific areas where to give more attention to improve the situation. Second, mixed model analysis of the clustered data appropriately fits the model parameters adjusting for the within cluster association and provides with valid inference.

The mixed model analysis of BDHS pooled data reveals that age has significant effect on women’s knowledge about HIV/AIDS. In the context of Bangladesh, women from different age groups differ in lifestyle, health practice, adaptability, maturity, accessibility, sex behaviors, etc., which could be the underlying reason behind the influence of women’s age on their knowledge about HIV/AIDS. The mixed model analysis also shows that the women aged 20–29 years are more likely to have knowledge about HIV/AIDS compared to young married women (15–19 years). Young married women often get limited exposure to sex-related issues and suffer from immaturity [21], which contribute to their vulnerability to HIV infection due to having little knowledge about HIV/AIDS [29]. Our finding is in agreement with other studies [10], reported earlier. In addition, we found women aged 30 years or above are less likely to have knowledge about HIV/AIDS. Conversely, Van Huy et al. [30] showed that women aged 30 years or above are more likely to have knowledge about HIV/AIDS, however, this study was in the context of Vietnam. The earlier studies in the context of Bangladesh, e.g. Hossain et al. [10], Yaya et al. [17], reported that the knowledge about HIV/AIDS among the women aged 30–49 years is lower (insignificant) compared to 15–19 years age group. In agreement with the direction of the association, this study demonstrates that the likelihood of knowledge among the women aged 30–49 is significantly lower in comparison with the women aged 15–19 years. The rationale of this finding is that married women aged 30 years or more are less adaptive to absorbing information compared to young women, which reduces their likelihood of knowing about HIV/AIDS.

The women’s knowledge about HIV/AIDS is found to vary significantly across the divisions of Bangladesh. Particularly, the women from Khulna and Rajshahi have the highest and lowest likelihood of having knowledge about HIV/AIDS, respectively, compared to Barisal division. Although Khan [21] reported that women from Dhaka division have the highest likelihood of HIV/AIDS knowledge, our finding is consistent with recent findings of Hossain et al. [10]. The divisions of Bangladesh possess inherent variabilities in terms of education, health, economy, media exposure, etc. that often contribute to the differences in women’s knowledge about HIV/AIDS at the division levels. Particularly, women from Khulna division have more access to media, more likely to use contraceptive methods than the women from Barisal division [31]. This could be the underlying reason, as these factors are found to be important for knowledge about HIV/AIDS among the women in Bangladesh. However, the low likelihood for the women at Rajshahi cannot be easily interpreted. As such, in BHDS 2011 and 2014, the Rangpur division has been introduced as a separate division that was once a part of Rajshahi division (in BDHS 2007), however, we merged Rajshahi and Rangpur division as Rajshahi in this study. The Rangpur division possesses low percentage of contraceptive user and high percentage of poor women [6] that might have confounded the effect of Rajshahi division compared to Sylhet division.

The type of residence has been found to be significantly associated with the knowledge of women about HIV/AIDS. Particularly, women living in urban areas are more likely to have knowledge about HIV/AIDS compared to rural women, which is in accordance with other studies [10, 21, 30, 32]. Contrary to our findings, there are few studies [17], which reported that the type of residence is unrelated to women’s knowledge about HIV/AIDS. However, rural women are often abandoned, neglected, and deprived of better health care facilities [33], and some recent studies [34] reported rural women as vulnerable to HIV infection due to their low level of knowledge about HIV/AIDS. On the other hand, urban women often enjoy better living provided with easier access to health information, media, healthcare facilities, etc., which reduces the likelihood of HIV infection subsequently. This study also shows the effect of residence type over the years on the women’s knowledge about HIV/AIDS. We observe that over the survey years, the likelihood of having knowledge about HIV/AIDS has been decreasing. Although the urban women show higher likelihood of knowledge, over time rural women shows significant improvement compared to urban women. We expect this finding will help the government and policy makers to plan for resident specific programs concerning to spread HIV/AIDS knowledge and to create awareness among the rural and urban women in Bangladesh.

There is a significant association between the women’s knowledge about HIV/AIDS and their religion. Muslim women are found to be more knowledgeable about HIV/AIDS compared to non-Muslim women in Bangladesh. The rationale for this finding is that the codes of Islam strongly prohibits multiple sex partner, extra marital affair, etc. and contributes to lower prevalence of HIV among the Muslim [35], which we expect the indirect reasons behind the higher likelihood of knowledge about HIV/AIDS among the Muslim women in Bangladesh. However, sex-related risk behaviors are practiced in the real world scenario disregarding the Islamic codes by the Muslims [36]. For instance, the migrant workers, especially, men often practice unsafe sex with non-spousal partner in the foreign country and pose a great threat of HIV/AIDS infection/transmission when they return back to Bangladesh and resume to unprotected sex with their spouses [37]. This provides counter evidence on the lower level of knowledge and awareness among the Muslim women in Bangladesh. Likewise, to support the claim of women’s religion on their knowledge about HIV/AIDS, there are insufficient literatures in the context of Bangladesh. Thus, we would interpret our finding with caution and would recommend in-depth analysis regarding the effect of religion on the knowledge about HIV/AIDS among the women in Bangladesh. This study results show that women from a higher level of socio-economic status (middle and rich) have a higher likelihood of HIV/AIDS knowledge compared to poor women, which is consistent with the studies [30]. The justification of the result is that women from upper socio-economic class have more access to modern amenities and health care facilities, which increase their likelihood of knowing about HIV/AIDS.

The use of contraceptive during sex has been proved to be beneficial for the protection from regulation of birth and HIV/AIDS [21], which also translates to awareness about the sex-related disease and health protection. This study reports that women who use contraceptive during sex are more likely to have knowledge about HIV/AIDS compared to the non-contraceptive user. This finding is in agreement with the other studies [21, 38]. Additionally, we found that the effect of contraceptive use on the knowledge is significantly different for different age groups and type of residence. Particularly, this study states that women aged 30–49 years who use contraceptive have a higher likelihood of knowledge about HIV/AIDS. This is due to the fact that younger women often face difficulty in obtaining contraceptive (e.g. condom) and knowing about it’s benefits and usage. Also, it is found that rural women who use contraceptive are less likely to have HIV/AIDS knowledge. This is because rural women often consider the use of contraceptive as a means of birth control by being ignorant of it’s uses as a protection from sex-related health problems, e.g. AIDS or HIV infection. Our finding indicates that rural women contraceptive user require extra attention and need to be educated about the benefit of using contraceptive to prevent HIV/AIDS.

Education plays a vital role in determining the social status of an individual and also, translates to a better job and more access to information [16]. Earlier literature [39] has already identified education as an alternate vaccine for the incurable disease-AIDS. Mwamwenda [39] reported that HIV/AIDS knowledge is positively associated with increasing level of the respondent’s and their husband’s education. A similar finding is found in our study, which is in line with other relevant studies [10, 21, 30, 32, 33]. Exposure to media has also been reported as an important factor associated with the knowledge of married women about HIV/AIDS. Television, radio, and newspaper are effective media to reach the general people, which communicate important messages in the form of music, news reports, dramas, movies, advertisements, etc. that can profoundly influence public knowledge about HIV/AIDS. Our study result shows that women who got media exposure have a higher likelihood of knowledge about HIV/AIDS. This finding is consistent with earlier studies [10, 16, 32, 34, 40]. Analysing the trend over the years, we found compared to the survey year 2007, significant improvement is observed in the level of knowledge among the women with media exposure in 2014 survey year. This finding might help the policy makers to plan for a longer version of media exposure so as to disseminate the knowledge about HIV/AIDS among the women in Bangladesh.

Moreover, family planning knowledge has been proved to be effective for preventing HIV transmission [41]. Exposure to family planning through media often provides information and services to women about pregnancy, sexually transmitted diseases including AIDS, which contribute to the improvement of women’s knowledge about HIV/AIDS. Accordingly, in this study, we found women who heard about family planning through media are more likely to have knowledge about HIV/AIDS. Also, we found the effect of family planning knowledge varies significantly over different age groups of the respondents. Family planning exposure has been found to be more effective among the women aged 20–29 years. The current working status has been an important indicator of women’s knowledge about HIV/AIDS. Working women compared to non-working women often get more chances to discuss HIV/AIDS related health issues with their respective co-workers. Additionally, workers are often provided with health care facilities (e.g. medical unit) that educate the workers on HIV/AIDS. Again, being in a community with other workers, global issues like HIV/AIDS spread faster, which altogether increase the likelihood of their knowledge about HIV/AIDS. This finding is in accordance with other studies [16].

Despite several strengths of the study, there are few limitations, which we would like to mention for future research works. This study focuses on the knowledge status of married women which is determined by whether the respondents have heard about HIV/AIDS. Although the women who are aware about HIV/AIDS could be considered as knowledgeable about HIV/AIDS, however, causal relationship cannot be ensured necessarily. Awareness about HIV/AIDS could be more than only knowing about the disease, for instance, awareness on transmission or awareness on prevention, etc., which is beyond the scope of this study. Although we have examined the influence of protective behaviours (e.g. condom use and family planning knowledge) and other covariates on the knowledge about HIV/AIDS, however, knowledge status of women could also influence their protective behaviour, which is beyond the scope of this study and not consistent with the specific objective. There is a possibility that the knowledge status of husbands influence the knowledge status of the respondents, however, we could not analyse knowledge information of husbands due to the unavailability in the BDHSs data. Analysing the effects of the determinants on the knowledge about HIV/AIDS among the women in Bangladesh could be more interesting if we could incorporate the information regarding HIV incidence. However, due to unavailability of incidence information in BDHS data, we could not elaborate our findings in relation to HIV incidence.

## Conclusion

In conclusion, this study analysed the pooled data from three most recent nationally representative surveys (BDHS: 2007, 2011, and 2014) to determine the trends and determinants of the women’s knowledge about HIV/AIDS in Bangladesh. Using a mixed modeling approach, this study reveals women’s age, region, residence, religion, socio-economic status, women’s and husbands’ highest level of education, media use, contraceptive use, family planning knowledge have a significant influence on women’s knowledge about HIV/AIDS. Additionally, we found that the rural women who use contraceptive are less likely to be knowledgeable about HIV/AIDS, which is a great concern as rural women despite using contraceptive possess little knowledge about HIV/AIDS. This study demonstrates that the effect of women’s age is confounded due to contraceptive use and family planning knowledge on explaining the likelihood of women’s knowledge about HIV/AIDS. Moreover, analysing the trend we found, over the years the likelihood of women being knowledgeable about HIV/AIDS has been decreasing, however, the likelihood has been observed to be increasing for rural women and media users, over the years. This study recommends residence specific programs to disseminate HIV/AIDS awareness effectively and also highlights the role of media exposure to create awareness about HIV/AIDS. Moreover, this study also recommends educating both the women and their husbands to have a greater impact on the knowledge about HIV/AIDS among the women in Bangladesh.
